# Radionuclide Imaging of Invasive Fungal Disease in Immunocompromised Hosts

**DOI:** 10.3390/diagnostics11112057

**Published:** 2021-11-06

**Authors:** Ismaheel O. Lawal, Kgomotso M. G. Mokoala, Mankgopo M. Kgatle, Rudi A. J. O. Dierckx, Andor W. J. M. Glaudemans, Mike M. Sathekge, Alfred O. Ankrah

**Affiliations:** 1 Department of Nuclear Medicine, University of Pretoria, Pretoria 0001, South Africa; ismaheellawal@gmail.com (I.O.L.); kgomotso.mokoala@up.ac.za (K.M.G.M.); mankgopo.kgatle@sanumeri.co.za (M.M.K.); mike.sathekge@up.ac.za (M.M.S.); 2 Nuclear Medicine Research Infrastructure (NuMeRI), Steve Biko Academic Hospital, Pretoria 0001, South Africa; 3 Medical Imaging Center, Department of Nuclear Medicine and Molecular Imaging, University Medical Center Groningen, 9700 RB Groningen, The Netherlands; r.a.dierckx@umcg.nl (R.A.J.O.D.); a.w.j.m.glaudemans@umcg.nl (A.W.J.M.G.); 4 National Center for Radiotherapy Oncology and Nuclear Medicine, Korle Bu Teaching Hospital, Accra GA-222 7974, Ghana

**Keywords:** radionuclide imaging, invasive fungal disease, immunosuppression, HIV, [^18^F]FDG PET/CT

## Abstract

Invasive fungal disease (IFD) leads to increased mortality, morbidity, and costs of treatment in patients with immunosuppressive conditions. The definitive diagnosis of IFD relies on the isolation of the causative fungal agents through microscopy, culture, or nucleic acid testing in tissue samples obtained from the sites of the disease. Biopsy is not always feasible or safe to be undertaken in immunocompromised hosts at risk of IFD. Noninvasive diagnostic techniques are, therefore, needed for the diagnosis and treatment response assessment of IFD. The available techniques that identify fungal-specific antigens in biological samples for diagnosing IFD have variable sensitivity and specificity. They also have limited utility in response assessment. Imaging has, therefore, been applied for the noninvasive detection of IFD. Morphologic imaging with computed tomography (CT) and magnetic resonance imaging (MRI) is the most applied technique. These techniques are neither sufficiently sensitive nor specific for the early diagnosis of IFD. Morphologic changes evaluated by CT and MRI occur later in the disease course and during recovery after successful treatment. These modalities may, therefore, not be ideal for early diagnosis and early response to therapy determination. Radionuclide imaging allows for targeting the host response to pathogenic fungi or specific structures of the pathogen itself. This makes radionuclide imaging techniques suitable for the early diagnosis and treatment response assessment of IFD. In this review, we aimed to discuss the interplay of host immunity, immunosuppression, and the occurrence of IFD. We also discuss the currently available radionuclide probes that have been evaluated in preclinical and clinical studies for their ability to detect IFD.

## 1. Introduction

Fungi are ubiquitous organisms found in soil and organic matter in all regions of the world. They occur as free-living organisms in the environment or as part of the normal flora of animals and humans. About five million fungi species have been identified, with less than 500 of them causing human infections [1,2]. Fungi gain access into the human body through the inhalation of aerosolized fungal conidia or the inoculation of fungal agents into deeper tissues during a traumatic injury or percutaneous medical procedure or the translocation of fungal agents following a bridge in mucosal integrity [1]. Most cases of human fungal infection do not lead to clinical disease due to efficient curtailment by the host immune defense. In immunocompromised hosts, fungal infection may become disseminated, causing life-threatening invasive fungal disease (IFD). Every year, IFD causes about 1.5 million deaths globally [3]. More than 90% of deaths from IFD are due to *Candida* sp., *Aspergillus* sp., *Cryptococcus* sp., and *Pneumocystis* sp. [3]. Fungi can exist as unicellular yeasts or as molds, which form branching hyphae [1]. Dimorphic fungi occur as molds in the environment and as yeast within human tissues.

There are many factors that drive the burden of IFD seen in contemporary medical practice. These factors include delayed recognition and diagnosis, the increasing rate of resistance to anti-fungal agents, and the increasing incidence of compromised host immunity as a side effect of medical therapies [4,5,6]. Several inherited and acquired conditions are known to cause immunosuppression predisposing to IFD. IFD occurring due to compromised host immunity has been best characterized in patients with hematologic malignancies, hematopoietic cell transplant and solid organ transplant recipients, patients with inherited immune dysfunctions, patients with human immunodeficiency (HIV) infection, and patients with prolonged neutropenia [7,8,9,10]. Other patients with an increased risk of IFD include those with chronic medical conditions associated with impaired immunity, such as uncontrolled diabetes mellitus, and critically ill patients requiring intensive care unit admission [11,12]. In recent times, an increased incidence of IFD has been reported in patients who are critically ill due to severe acute respiratory syndrome coronavirus-2 (SARS-CoV-2) infection [13,14].

Definitive diagnosis of IFD requires histopathological examination and/or culture of a sterile specimen obtained from the infection site [15]. Biopsy is not always feasible because the site of fungal infection is unknown, or the procedure is considered unsafe due to the severity of the underlying illness or risk of bleeding. Bronchoalveolar lavage is the standard clinical procedure for obtaining respiratory samples to confirm the etiology of respiratory disease including IFD involving the lungs. Several noninvasive rapid molecular tests have been evaluated for their sensitivity and specificity in diagnosing IFD and monitoring the response to antifungal therapy [16]. Many factors still affect the performance of these non-culture-based techniques, including variability in diagnostic performance, poor diagnostic utility in patients already on antifungal therapy, and limited utility for response assessment [17,18]. Imaging with computed tomography (CT) and magnetic resonance imaging (MRI) has been recommended as an ancillary tool in diagnosing IFD. These morphologic imaging modalities rely on tissue architectural changes for the diagnosis of IFD. Their diagnostic performance is limited by the delayed appearance of these tissue changes, the lack of specificity of the imaging findings for IFD, and the variability in the appearance of different types of IFD on morphologic imaging [19,20,21]. Improvement in morphological tissue architectural distortions caused by IFD trail behind the microbiological response, making these imaging techniques unsuitable for early response assessment in treated patients. Radionuclide imaging techniques with positron-emission tomography (PET) or single-photon emission computed tomography (SPECT) target the pathogen that causes the disease or host immune response in infection imaging [22]. The direct targeting of pathogenic fungal organisms has the potential for IFD diagnosis with high specificity and may be useful for treatment response assessment [23]. There is evidence showing a superior diagnostic performance for fluorine-18 fluorodeoxyglucose ([^18^F]FDG) PET/CT over morphologic imaging with stand-alone CT in patients with IFD [24,25]. Novel radiopharmaceuticals targeting different metabolic pathways or molecular structures of pathogenic fungi are also in the pipeline for clinical translation [26]. In this review article, we aim to summarize the interplay of host immunity, immunodeficiency states, and the occurrence of IFD. We will also discuss the utility of radionuclide imaging techniques in diagnosing and managing IFD in the immunocompromised host using radiopharmaceuticals that target host immune response and the causative pathogen. We will conclude by providing insights into factors that must be considered in broadening the application of radionuclide imaging techniques for IFD.

## 2. Host Immunity, Immunodeficiency, and Invasive Fungal Disease

Multiple layers of host immune defenses are present to protect against IFD. Some of the pathogenic fungal species causing infection in humans are present as commensals within the human body. Fungal agents existing as commensals within the immunocompetent host may become pathogenic, causing opportunistic disease (IFD) in the immunocompromised host [27,28]. Several fungal factors also play prominent roles in driving the conversion of colonization to invasive disease, including fungal virulence factors and morphology (yeast versus hyphal form) [29,30].

### 2.1. Host Immunity against Invasive Fungal Disease

The innate and adaptive immune responses play critical roles against the dissemination of fungi in the body. Innate immunity represents the first line of defense against invasive fungal infection. The physical barrier created by the skin and the mucosal surfaces prevents the translocation of the fungal agent into deeper tissues. Candidalysin is a cytolytic peptide toxin produced by *Candida albicans* [31]. Candidalysin disrupts mucosal integrity, leading to the invasion of the host tissue by *Candida albicans*. The mucociliary escalator system of the respiratory tract also serves to clear inhaled fungal conidia from the respiratory epithelium. The mucosal barrier integrity of the respiratory epithelium is compromised in individuals with chronic pulmonary disorders such as chronic obstructive pulmonary disorder, bronchial asthma, and alpha-1 anti-trypsin deficiency, predisposing them to pulmonary fungal infections [32,33].

Innate immunity is the immediate non-specific body response to pathogenic organisms, including fungi. The host innate immune response to pathogenic fungi consists of cellular and humoral components. The humoral component of the innate immunity against invasive fungal infection includes various soluble factors, including alarmins, different antimicrobial peptides, and the complement system. Alarmins, danger-associated molecular patterns (DAMPs), are constitutively expressed soluble factors released by damaged tissues during infections. They act as chemotactic and immune-activating factors [34]. Antimicrobial peptides (AMPs) that constitute part of the humoral component of the innate immunity against invasive fungal infection include defensins, LL-37, cathelicidin (hCAP-18), histatin 5, serprocidin, and lysozyme [35,36,37,38]. AMPs exert antifungal activity by attacking the fungal cell membrane, cell wall, or intracellular targets to cause cellular destruction via osmotic damage. Complement components playing a crucial role in the body’s defense against fungal disease include C3a and C5a (anaphylatoxins/chemoattractants that recruit phagocytic cells), C3b/iC3b (opsonin that promotes phagocytosis), and C5b-9 (membrane attack complex or terminal complement complex that causes lysis of pathogen) [39].

**Figure 1:**
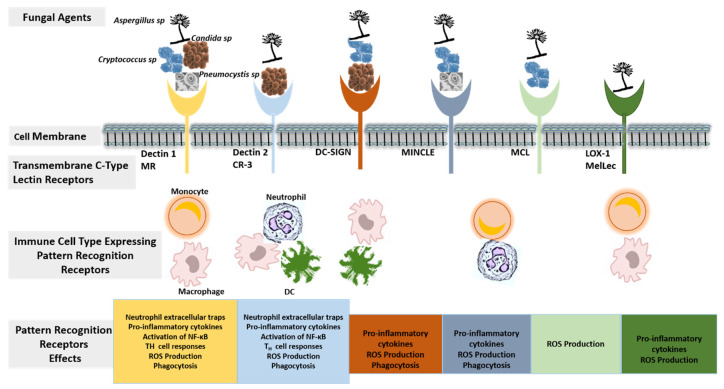
A schematic diagram showing the components of host innate immunity during interaction with fungal agents. Several transmembrane C-type lectin receptors including dectin-1, dectin-2, mannose receptor (MR), complement receptor-3 (CR-3), dendritic cell-specific intercellular adhesion molecule-3-grabbing nonintegrin (DC-SIGN), macrophage inducible C-type lectin (MINCLE), macrophage C-type lectin (MCL), and lectin-type oxidized low-density lipoprotein receptor 1 (LOX-1) are expressed on the cell surface of immune cells such as monocytes/macrophages, neutrophils, and dendritic cells (DC) and serve as important components of the pattern-recognition receptors (PRRs) that recognize invading fungi agents. Following the recognition of pathogen-associated molecular patterns (PAMPs) of the fungus by the immune cells through their PRRs, multiple anti-fungal activities are triggered downstream, including the production of neutrophil extracellular traps by neutrophils, the production of proinflammatory cytokines such as nuclear factor kappa B (NF-kB), the production of reactive oxygen species (ROS), the activation of adaptive immune response, and the phagocytosis of fungal organisms.

The cells of the innate immunity participating in the host response against fungal disease include macrophages, dendritic cells, polymorphonuclear cells, natural killer cells, and myeloid-derived suppressor cells [2]. The interaction between the fungal pathogen-associated molecular patterns (PAMPs) and pathogen recognition receptors (PRRs) expressed by immune cells is germane to activating the host innate immune system against fungal disease (Figure 1). PAMPs are cell wall components of fungi and are shared by fungi belonging to different genera. The best characterized PAMP molecules are α- and β-glucan, N- and O-linked mannans, lipopolysaccharides, peptidoglycan-associated proteins, and phospholipomannan [2,40]. PRRs are expressed by innate immune cells (macrophages, dendritic cells, and polymorphonuclear phagocytes), adaptive immune cells (B and T lymphocytes), and non-immune cells (epithelial cells and fibroblasts). The most characterized PRRs participating in antifungal host immune activity belong to the Toll-like receptors (TLRs), C-type lectin receptors (CLRs), retinoic acid-inducible gene 1-like receptors (RLRs), and nucleotide-binding oligomerization domain-like receptors (NLRs) [41,42].

Macrophages are important phagocytic and antigen-presenting cells. They fulfil the important roles of engulfing the invading fungi and function as the link between innate and adaptive immunity. In *Aspergillus fumigatus* infection, macrophages inhibit fungal spore germination by forming dense clusters around the spores [43]. Macrophage phagocytosis of fungus into the phagosome is followed by phagosome–lysosome fusion that triggers the generation and release of antimicrobial agents that destroy the fungus [44]. Other macrophages secrete cytokines that attract other inflammatory cells to the infection site [45]. Macrophages, along with other inflammatory cells, form granulomas, which are typical in some fungal infections including cryptococcosis [46]. Like macrophages, dendritic cells (DCs) are antigen-presenting cells but are less efficient in pathogen killing [47]. DCs digest the antigen and present it to naïve T cells, causing their differentiation into different T-helper subsets, including T-helper 1 (Th1) and T-helper-17 (Th17), both of which play critical roles in immunity against fungal disease [48,49]. Neutrophils are the most abundant of polymorphonuclear cells and the most important for innate antifungal immunity. This underscores the role of neutropenia in the predisposition to IFD [50]. The antifungal property of neutrophils relates to their ability to produce chemokines that are chemoattractants aiding the chemotaxis of inflammatory cells to the infection site and soluble factors with antimicrobial, proteolytic, and nucleolytic properties that damage pathogenic fungi. The hyphal form of fungi is the tissue-invading phenotype. Fungal hyphae may be too large for phagocytosis. Neutrophils produce neutrophil extracellular traps (NET) for the extracellular trapping and killing of fungal hyphae [51]. Natural killer (NK) cells are large lymphocytes that participate in host innate immunity. NK cells cause cytotoxicity by utilizing perforin and granzyme. NK cells also produce cytokines that regulate the function of other immune cells [52]. The antifungal function of NK cells occurs via the damage of fungal hyphae, as seen in infections due to *Candida albicans* and *Aspergillus fumigatus* [53].

Acquired immunity provides a slower but more specific antifungal immune response via T-cell-mediated cytotoxicity and B-cell-mediated humoral immunity. T cells are broadly classified as helper CD4 T cells or cytotoxic CD8 T cells. CD 4 T cells play a regulatory role by producing cytokines that drive the recruitment of phagocytic cells to the site of infection [54]. The activation of CD4 T cells causes their differentiation into the various subsets of T helper cells, each subset producing unique sets of cytokines [55]. Balanced Th1 and Th17 responses have vital antifungal properties through the production of cytokines such as tumor necrosis factor-alpha (TNF-α), interferon-gamma (IFN-γ), and interleukin (IL)-17 that drive phagocytic fungal clearance [48]. The antifungal properties of CD8 T cells occur via the direct killing of pathogenic fungal cells and lysis of fungal-infected host cells. B cells are responsible for the humoral arm of adaptive immunity. The hallmark of B cell activation is the production of antibodies with antifungal properties, including the prevention of fungal entry, inhibition of fungal replication, modulation of the other arms of host immunity, suppression of fungal release of polysaccharide and germ tube formation, neutralization of fungal-produced toxins, and the inhibition of biofilm production [2,56].

### 2.2. Immunodeficiency States and Invasive Fungal Disease

Advances in medical knowledge, rather than contributing to reducing the morbidity and mortality of IFD across different risk groups, have contributed to the burgeoning list of conditions causing immunodeficiency, particularly related to novel therapies with deleterious effects on host immunity [57]. Several disease states are known to be associated with some levels of immune dysfunction. This section will briefly discuss the immune dysfunction predisposing to IFD for the few most important groups of immunocompromised hosts. The discussion presented in this section is by no means exhaustive. Only a summary of the important causes of immunosuppressed states that predispose to IFD is presented.

Primary immunodeficiencies are a group of rare inborn errors of immunity. Inherited immunodeficiency syndromes causing severe combined immunodeficiencies or those that impair the phagocytic function of the immune cells predispose to opportunistic fungal diseases, including IFD. Two prototypic primary immunodeficiency conditions predisposing to opportunistic fungal diseases, chronic granulomatous disease due to mutations in the subunits of NADPH and myeloperoxidase deficiency, provided the earliest insights into the role of defective phagocytic oxidative machinery in the predisposition to opportunistic fungal disease [1,58]. More recently, primary immunodeficiency resulting from alterations in the IL-12/IFN-γ and JAK/STAT signaling pathways has been characterized [9,59]. The list of primary immunodeficiency conditions predisposing to IFD is growing with advances in molecular techniques [59,60]. A detailed discussion on this subject is beyond the scope of this present work but has been recently reviewed by others [1,9,61,62].

Acquired immunodeficiencies are more common predisposing factors to IFD. The most common acquired causes of immunodeficiency states that predispose to IFD include hematopoietic cell transplantation, hematologic malignancies, solid organ transplantation, prolonged neutropenia (absolute neutrophil counts of <500 cells/µL lasting more than ten days) from any cause including chemotherapy and immunosuppressive therapies, and advanced HIV infection [63,64].

Hematopoietic cell transplantation (HCT) is utilized to treat various clinical conditions, including neoplastic, inflammatory, autoimmune, and genetic diseases [65,66]. In the treatment of hematologic malignancies, immunocompetent donor cells recognize and destroy host cancer cells. However, the immunocompetent donor cells may also identify incompatible HLA (human leukocyte antigen) expressed by the host cells and mount immune attacks against them, leading to graft-versus-host disease (GvHD). Several factors are prevailing in patients with hematological malignancies that are treated with HCT that predispose to IFD, including prior exposure to cytotoxic therapies, immunosuppressive therapy to prevent or treat GvHD, prior infection or colonization by pathogenic fungi, mucosal barrier disruption (especially as a component of GvHD), and metabolic alterations (such as diabetes mellitus, chronic liver disease, malnutrition, and iron overload) [67,68]. All these factors work in concert to cause immunosuppression in the host with an attendant increased risk of IFD [67]. The annual incidence of IFD in HCT recipients ranges between 3.4% and 8.8% [69,70]. The most common IFD types in HCT recipients are invasive aspergillosis (43% to 81%), invasive candidiasis (11% to 28%), and zygomycosis (4% to 8%) [69,70]. Of all cases of invasive aspergillosis, *Aspergillus fumigatus* is the causative agent in about 44% of HCT recipients [69].

Like in HCT recipients, solid organ transplant (SOT) recipients also experience immunosuppression resulting from immunosuppressive therapy to prevent organ rejection. Risk factors for IFD in SOT recipients include complicated surgery or repeat surgery, pathogenic fungi colonization of the transplanted organ, graft rejection, and prolonged immunosuppressive therapy [71]. The incidence of IFD in the first 12 months after SOT is 3.1% [8,72]. The most common form of IFD in SOT recipients is candidiasis, accounting for about half of all cases [71]. Other forms of IFD in SOT recipients are invasive aspergillosis, cryptococcosis, non-aspergillus invasive molds disease, and endemic fungi such as histoplasmosis, coccidioidomycosis, and blastomycosis [8].

Immunosuppression is the desired effect in treating conditions such as autoimmune disease and an off-target effect in treating disorders such as malignant disease. Ibrutinib is a tyrosine kinase inhibitor that has shown remarkable success in treating lymphoid malignancies such as mantle cell lymphoma, chronic lymphocytic leukemia, Waldenström macroglobulinemia, diffuse large B cell lymphoma, and primary CNS lymphoma [73,74,75]. Ibrutinib is an irreversible inhibitor of Bruton tyrosine kinase (BTK). BTK is present in immune cells, including B cells, neutrophils, monocytes, and macrophages, where it mediates both innate and acquired immune function. Therefore, the inhibition of BTK in patients receiving ibrutinib for lymphoid malignancies is associated with serious infectious complications, including IFD [76]. The striking difference between IFD complicating ibrutinib therapy versus IFD occurring in HCT or SOT recipients is that IFD occurs in the former without neutropenia, lymphopenia, or corticosteroid use. This observation reflects qualitative, rather than quantitative, defects in immune cells [76]. Organisms causing IFD in ibrutinib-treated patients are *Pneumocystis jirovecii*, *Cryptococcus neoformans*, and filamentous fungi, including *Aspergillus*, *Fusarium*, and *Mucorales* [77,78].

In the early 1980s, an epidemic of *Pneumocystis jirovecii* pneumonia (PJP) heralded the acquired immunodeficiency syndrome (AIDS) pandemic [79]. Human immunodeficiency virus (HIV), the causative agent of AIDS, utilizes CD4 molecules expressed on T-helper cells and other immune cells (including macrophages and dendritic cells) to infect and destroy the immune cells [80]. This targeting of immune cells leads to generalized immunosuppression in severe HIV infection. Immune functions impaired in HIV infection include decreased production of IFN-γ, impaired phagocytosis by macrophages, impaired chemotaxis and oxidative killing by neutrophils, and decreased B cell antigen responsiveness [81]. Despite the widespread availability of effective antiretroviral therapy and early testing for HIV infection, both of which have led to a decline in the prevalence of severe immunosuppression in HIV-infected patients, IFD continues to be a significant driver of mortality among people living with HIV infection. IFD causes about 1 million deaths annually, accounting for 50% of AIDS-related mortality [82]. The most important forms of IFD in people living with HIV infection include PJP, candidiasis, cryptococcoses, histoplasmosis, coccidioidomycosis, talaromycosis, penicilliosis, and aspergillosis [80,81,82,83].

## 3. Radionuclide Imaging of Invasive Fungal Disease

**Figure 2:**
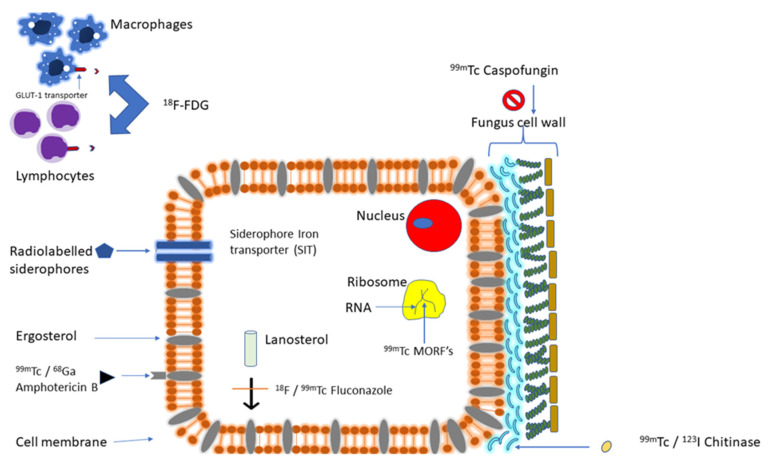
A schematic diagram of the fungal cell and surrounding inflammatory cells (macrophages and lymphocytes). [^18^F]FDG is mostly taken up by host inflammatory cells that are abundantly present at the sites of invasive fungal disease. Radiolabeled siderophores produced by ex vivo labeling of synthetic siderophores or in vivo labeling of fungal-produced siderophores following administration of radiogallium are trapped by the fungal cell via siderophore–iron transporter expressed in the fungal cell membrane. Fluconazole, amphotericin, and caspofungin are anti-fungal agents that have been radiolabeled for specific targeting of fungal agents in IFD. Radiolabeled chitinase targets the fungal cell wall in fungal-specific imaging. Morpholino oligomers (MORFs) target fungal messenger ribonucleic acid (RNA) and provide a specific means of targeting fungal organisms.

Radionuclide imaging utilizes radiopharmaceuticals targeting the host response or specific molecular pathways or structures within the pathogen [22]. Host immune response is an early process in the disease course. Targeting host immune response to pathogenic fungi causing IFD, therefore, offers an opportunity for the early detection of IFD. Different radiopharmaceuticals targeting various molecular structures or pathways of fungi pathogenic to humans are in the developmental pipeline. Targeting fungi causing IFD offers an opportunity for more specific detection of IFD and the ability to confirm fungal clearance following successful antifungal therapy. Radionuclide imaging is routinely whole-body, allowing the quantification of the whole-body burden of IFD, a piece of information that may have therapeutic implications. This section will discuss the radionuclides that target host immune response or fungi-specific molecular pathways or structures that have been evaluated in preclinical and clinical studies for SPECT and PET imaging of IFD (Figure 2).

### 3.1. Targeting Host Immune Response

Following tissue invasion by pathogenic fungi such as Cryptococcal species, the host mounts an immune response leading to the formation of granulomas [84]. A granuloma consists of inflammatory cells, including macrophages, dendritic cells, T cells, and B cells surrounding a necrotic core [2]. The granuloma creates a milieu that brings T cells and B cells close to macrophages to allow for their activation. The accumulated inflammatory cells confine the killing zone around pathogenic organisms and prevent the spillage of toxic metabolites into the systemic circulation [2]. In the immunocompetent host, the granuloma is efficient in curtailing the growth of the pathogenic organism.

Inflammatory cells, especially macrophages and lymphocytes, utilize glucose for metabolism. The rate of glucose utilization is accentuated by immune cell activation during inflammation and infection. [^18^F]FDG is a radioactive analogue of glucose and the most used radiopharmaceutical for PET imaging of infection. Among all radiopharmaceuticals for radionuclide imaging of IFD, [^18^F]FDG for PET imaging has the most robust evidence regarding its utility in the initial assessment and treatment response assessment of IFD in immunocompromised patients.

Early studies evaluating the utility of [^18^F]FDG PET/CT in IFD imaging were limited to retrospective case reports and case series [85,86,87,88,89]. In one early study by Hot et al. that utilized [^18^F]FDG with PET-only in immunocompromised patients with proven or probable IFD, [^18^F]FDG PET detected all sites of IFD involvement previously identified on conventional CT and MRI in all patients imaged for the initial assessment of IFD [90]. In addition, among ten patients with disseminated candidiasis, [^18^F]FDG PET detected sites of IFD involvement not discernible on CT in six patients [90]. These early studies provided the earliest evidence regarding the ability of [^18^F]FDG PET to detect fungal lesions. In addition, and despite the limitation of PET-only technology without anatomical correlation with CT, a superior lesion detection rate was reported for [^18^F]FDG PET than conventional imaging with stand-alone CT or MRI [90]. Despite this higher diagnostic sensitivity, the limitation of the PET-only technology must be emphasized, especially regarding the difficulty with the differentiation of pathologic [^18^F]FDG uptake due to disease from physiologic [^18^F]FDG uptake. Additionally, the lack of anatomic correlation precludes the accurate localization of IFD to the organ of involvement.

In recent times, larger studies have reported the diagnostic utility of [^18^F]FDG PET/CT in the initial evaluation and treatment response assessments of immunocompromised hosts with proven, probable, or possible IFD [26,91]. A recent study by Ankrah et al. has provided insights into the relative lesion detection rates of [^18^F]FDG PET/CT versus morphologic imaging with X-ray, CT, MRI, or ultrasound [92]. The authors compared the findings on 121 [^18^F]FDG PET/CT scans with 216 morphologic imaging studies obtained within two weeks of [^18^F]FDG PET/CT in a group of immunocompromised patients evaluated for different indications. Findings on [^18^F]FDG PET/CT and morphologic imaging were concordant in 109 of 121 (90%) [^18^F]FDG PET/CT scans. As expected, [^18^F]FDG PET/CT detected more pulmonary lesions in 6 of 80 chest radiographs performed to evaluate pulmonary IFD. Additionally, [^18^F]FDG PET/CT scan detected more lesions in 3 of 33 ultrasounds scans. In 14 diffusion-weighted MRIs performed to assess intracerebral IFD, [^18^F]FDG PET/CT failed to detect disease in three studies. The study by Ankrah et al. also showed the added value of whole-body imaging with [^18^F]FDG PET/CT compared with region-based morphologic imaging [92]. In a significant proportion of patients (about 50% of studies), [^18^F]FDG PET/CT detected lesions outside the body region imaged on morphologic imaging with X-ray, CT, MRI, or ultrasound. Morphologic imaging with CT and/or MRI is the current recommended imaging modality for assessing IFD [5,15]. In the study by Ankrah et al., morphologic imaging with stand-alone CT was concordant with [^18^F]FDG PET/CT for assessing the pulmonary involvement of IFD [92]. The whole-body imaging afforded by [^18^F]FDG PET/CT led to the detection of extra-pulmonary lesions compared with high-resolution chest CT. The high physiologic brain uptake of [^18^F]FDG suggests that [^18^F]FDG PET/CT is not sufficient for assessing brain lesions, especially when those lesions are subtle or are not intensely avid for the radiopharmaceutical.

Douglas and colleagues have also evaluated the diagnostic performance of [^18^F]FDG PET/CT compared with diagnostic CT in the assessment of 45 immunocompromised patients with 48 episodes of proven or probable IFD [70]. In this study, unlike with the study by Ankrah et al. [92], the authors reported a better pulmonary lesion detection rate for [^18^F]FDG PET/CT than diagnostic CT mainly due to the more definite focal areas of [^18^F]FDG avidity in pulmonary nodules suggestive of pulmonary IFD compared with non-specific consolidation seen on stand-alone CT [93]. [^18^F]FDG PET/CT detected clinically occult disease in 40% of patients and IFD dissemination to extra-pulmonary sites in 38% of cases. Extra-pulmonary sites of IFD involvement seen on [^18^F]FDG PET/CT but not on stand-alone CT were intraabdominal (hepatic, splenic, and intra-abdominal collection in three patients), musculoskeletal (bone and muscle involvement in two patients), and brain and orbital involvement in one patient [93]. Interestingly, 18% of all cases of IFD reported in this study were incidental findings on [^18^F]FDG PET/CT scan acquired for other indications. This calls for a consideration of IFD in the differential diagnosis of [^18^F]FDG-avid lesions on PET/CT performed in immunocompromised patients imaged for different indications other than the assessment of IFD. The results from the studies by Ankrah et al. and Douglas et al., in combination, suggest that while both [^18^F]FDG PET/CT and stand-alone CT have a similar detection rate for lung involvement in IFD, a performance primarily driven by CT even as hybrid [^18^F]FDG PET/CT, findings on [^18^F]FDG PET/CT are more easily ascribable to IFD compared with the non-specific findings on stand-alone CT [92,93]. Consistently, both studies show the superiority of [^18^F]FDG PET/CT over stand-alone CT in detecting extra-pulmonary sites of involvement—information that may have therapeutic implications and affect treatment outcome.

[^18^F]FDG PET/CT imaging findings are not always positive in all cases of IFD. Apart from its suboptimal performance compared to MRI in assessing intra-cerebral IFD, candidemia without specific organ involvement results in false-negative [^18^F]FDG PET/CT scans [94]. In a retrospective study of 51 immunosuppressed patients, including 29 patients (18 with proven and 11 with suspected IFD) imaged for the initial assessment for IFD, Leroy-Freschini and colleagues reported a diagnostic accuracy of 93% for [^18^F]FDG PET/CT when used in the initial assessment of patients with proven or suspected IFD [94]. False-negative findings in this study were due to candidemia without specific organ involvement seen in two patients. In 19 of the 29 patients, morphologic imaging was acquired before [^18^F]FDG PET/CT. Findings on [^18^F]FDG PET/CT and morphologic imaging were concordant in nine patients (two negative and seven positive findings) and discordant in 10 patients. In all discordant patients, [^18^F]FDG PET/CT outperformed morphologic imaging with CT or MRI by being more accurate in determining the extent of disease involvement in an organ (*n* = 3) or determining other sites of IFD dissemination (*n* = 7). [^18^F]FDG PET/CT failed to identify cerebral aspergillosis in one patient, seen on a prior MRI [94].

Beyond its use in the initial assessment of IFD, [^18^F]FDG PET/CT has found a greater application in the therapy response assessment of patients with IFD. This latter indication represents an area with a significant clinical need for different reasons. The duration of treatment of IFD with antifungal agents is not standardized but is typically long, usually lasting several months. This long duration of administration of expensive medications comes with an economic cost at a time of dwindling health budgets and competing health spending. Additionally, the long duration of antifungal therapy is associated with an increased risk of treatment-induced toxicity and treatment non-adherence. Morphologic imaging with CT and MRI is less suitable for therapy response assessment as tissue reparative changes trail off after successful pathogen clearance. Some studies have demonstrated the utility of [^18^F]FDG PET/CT as a noninvasive biomarker for treatment response assessment in patients on antifungal therapy for IFD [92,93,94,95].

Quantitative metrics derivable from [^18^F]FDG PET, including standardized uptake value (SUV), metabolic tumor/lesion volume (MTV), and total lesion glycolysis (TLG), have been applied for quantifying disease burden in different tumors [96,97,98,99,100]. These quantitative parameters are significant predictors of treatment outcome and survival in different cancers [101]. Ankrah and colleagues applied these metabolic metrics obtained on baseline [^18^F]FDG PET/CT for the initial assessment of IFD in immunocompromised patients [95]. The authors found that the baseline TLG and metabolic volume (MV) of lesions due to IFD are suitable to predict patients who achieve complete metabolic response on antifungal therapy. Using receiver operative characteristic (ROC) analysis, a TLG of 160 had an accuracy (area under the curve) of 95%, a sensitivity of 94%, and specificity of 100% in predicting patients who will achieve complete metabolic response to therapy [95]. MV obtained from baseline [^18^F]FDG PET/CT was also found suitable for predicting responders who achieved complete metabolic response to antifungal therapy versus non-responders with an accuracy of 91%.

**Figure 3:**
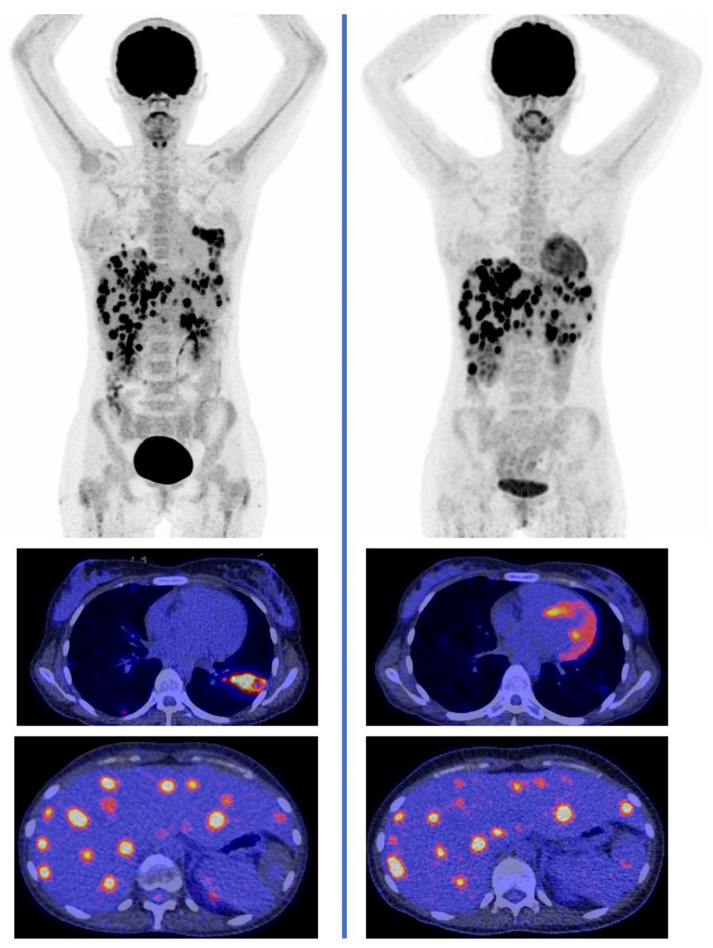
A 31-year-old female diagnosed with disseminated candidiasis after chemotherapy for acute lymphocytic leukemia. Baseline [^18^F]FDG PET/CT (**left** column) showed disease involvement in the lungs, liver, and spleen. Repeat [^18^F]FDG PET/CT after 3 months of voriconazole and caspofungin (**right** column) for treatment response assessment showed resolution of the lung lesions but persistence of the hepato-splenic lesions. Hepatosplenic candidiasis at baseline and after 3 months of therapy. The imaging finding led to a change in drug treatment.

By far, the most important added value of [^18^F]FDG PET/CT in patients on antifungal therapy is the ability to guide the duration of treatment. In most instances, treatment can safely be discontinued in patients who achieve complete metabolic response to therapy even if anatomic distortion due to IFD remains on morphologic imaging [95]. In patients who show disease progression evident by an increasing number, extent, and intensity of [^18^F]FDG-avidity in IFD lesions, a prolongation or change in treatment strategy may be warranted (Figure 3). A challenge to bear in mind here is the lack of specificity of [^18^F]FDG for fungal lesions. In typical immunocompromised patients at risk for IFD, other diseases with [^18^F]FDG-avid lesions, including non-fungal infections such as bacterial and viral opportunistic infections, malignancies, and inflammatory disorders, may be present, complicating image interpretation [92,102]. In such instances, it becomes imperative to distinguish between the progression of IFD versus co-existing non-fungal opportunistic infections or malignancies, especially in the context of new lesions appearing on follow-up [^18^F]FDG PET/CT in patients on antifungal therapy. The third scenario that can be encountered on [^18^F]FDG PET/CT for the treatment response assessment of IFD is a partial response or stable disease in which the appearance of lesions remains the same or has improved but has not resolved completely compared to previous studies [94,95]. This imaging phenotype may represent residual disease requiring the continuation of antifungal therapy or residual inflammation in patients with complete fungal clearance. At the time of discontinuation of treatment, there may be residual [^18^F]FDG avidity at the sites of IFD in patients who go on to have complete metabolic response without further antifungal therapy [95]. This phenomenon, which has been better characterized in patients treated for tuberculosis [103,104], is believed to result from ongoing host inflammatory response to dormant fungi whose replication has been curtailed by the host immune system or fungal antigens from dead organisms that the host immune system has not successfully cleared. A need, therefore, exists to identify [^18^F]FDG PET metrics capable of distinguishing residual disease needing further treatment from post-treatment inflammatory changes not requiring further treatment.

### 3.2. Targeting Fungal Molecular Structure or Pathway

Radionuclide imaging allows the noninvasive interrogation of molecular targets expressed by the host or the pathogen. [^18^F]FDG PET/CT is the radionuclide technique with the most robust evidence with its use. This is so despite the limitations associated with its application, including its non-specificity and the difficulty in differentiating post-treatment inflammation from residual IFD in patients on antifungal therapy. Direct targeting of the molecular structure or metabolic pathway expressed exclusively by the invading fungi has the potential to overcome the limitations associated with [^18^F]FDG PET/CT. In this section, we will discuss the radiopharmaceuticals that have been evaluated for specific pathogen targeting in IFD. We will discuss the promises and limitations of each radiopharmaceutical.

#### 3.2.1. Targeting Fungal Iron Utilization

Iron is an essential element for microbial growth. Iron, in humans, is not readily available for microbial use as it is sequestered in proteins such as ferritin, lactoferrin, and transferrin [105]. To acquire iron for their growth, pathogens such as fungi produce siderophores, which can extract iron from iron-containing proteins of the host [106]. Once it extracts iron, the siderophore–iron complex is taken up by the fungi via the siderophore–iron transporter (SIT) in an energy-dependent process. The allure of siderophore-based imaging lies in the upregulation of SIT by the fungi during infection [107], the exclusivity of SIT expression in the fungi and not in mammalian cells, the energy-dependent uptake of the siderophore–iron complex by SIT that ensures trapping only by viable fungi, and the low molecular mass of siderophores that ensures prompt uptake at the sites of infection and rapid renal elimination, leading to a good signal-to-noise ratio following in vivo administration of radiolabeled siderophores [108]. For radiolabeling, the ferric iron in siderophores can be easily substituted by iron-like radionuclides such as Gallium-68 and Zirconium-89 for PET imaging. Comprehensive reviews of siderophore-based imaging of fungal infection have been recently published [108,109].

Gallium-67 (^67^Ga) citrate, a SPECT tracer, was probably the first radiopharmaceutical exploring iron utilization by pathogens used for the clinical imaging of IFD. One of the proposed mechanisms by which [^67^Ga]Ga-citrate localizes to the infection site was by in vivo binding to pathogen-produced siderophores followed by subsequent uptake into the organism via SIT. Before the widespread availability of PET, [^67^Ga]Ga-citrate imaging was commonly applied for infection and oncology imaging. *Pneumocystis jirovecii* pneumonia (PJP), a leading opportunistic infection in advanced HIV infection, causes diffuse [^67^Ga]Ga-citrate uptake in the lungs [110,111]. [^67^Ga]Ga-citrate has better sensitivity than chest radiographs in the evaluation of PJP. [^67^Ga]Ga-citrate imaging in the right setting has an excellent negative predictive value for PJP [112]. Lung uptake of [^67^Ga]Ga-citrate is not specific for PJP as other prevalent entities in the immunocompromised host may also show avidity for [^67^Ga]Ga-citrate. These entities include cytomegalovirus infection, other fungal infections including histoplasmosis and cryptococcosis, bleomycin toxicity following chemotherapy, tuberculosis, and toxoplasmosis [110]. [^67^Ga]Ga-citrate has fallen out of favor due to its suboptimal image quality, high radiation burden on patients, the requirement for late imaging up to 48 to 72 h post tracer injection, and the availability of newer radiopharmaceuticals and PET technology with superior diagnostic performance. Gallium-68 (^68^Ga) citrate is a PET congener of [^67^Ga]Ga-citrate with superior diagnostic performance. [^68^Ga]Ga-citrate PET/CT has the potential to complement [^18^F]FDG PET/CT assessment of IFD since the former has striking differences in its biodistribution, allowing for a more robust assessment of disease involvement in regions of the body with high physiologic [^18^F]FDG uptake, such as the brain [113]. To date, no study has evaluated the possible role of [^68^Ga]Ga-citrate PET/CT in IFD.

There has been an advancement in the molecular targeting of fungal iron utilization for radionuclide imaging of IFD. In the pivotal work by Petrik and colleagues, the authors reported the successful labeling of two *Aspergillus fumigatus* siderophores (desferri-triacetylfusarinine C, TAFC and desferri-ferricrocin, FC) to ^68^Ga [114]. The complexes were stable in human serum and demonstrated uptake dependent on mycelia load, suggesting a potential utility for treatment response assessment. In an in vivo study with non-infected mice, [^68^Ga]Ga-TAFC showed rapid renal excretion with prompt background activity clearance while [^68^Ga]Ga-FC demonstrated high retention. In *Aspergillus fumigatus*-infected mice, [^68^Ga]Ga-TAFC showed lung uptake that depended on the severity of infection [114]. In a subsequent study by the same group, a broader array of *Aspergillus fumigatus* siderophores were similarly evaluated for their utility for imaging IFD [115]. Among the ^68^Ga-labeled siderophores tested, only [^68^Ga]Ga-TAFC and [^68^Ga]Ga-FOXE demonstrated sufficient stability in human serum and other reaction media. Both [^68^Ga]Ga-TAFC and [^68^Ga]Ga-ferrioxamine E (FOXE) demonstrated prompt renal excretion with barely any significant retention in any other organ in non-infected mice [115]. This finding suggests that these radiolabeled siderophores may be useful for imaging IFD involving all organs other than the kidneys. A common drawback of many radiopharmaceuticals is their lack of specificity. Petrik and colleagues evaluated the specificity of [^68^Ga]Ga-TAFC and [^68^Ga]Ga-FOXE for fungal disease [115]. Both complexes showed no significant uptake in bacterial (*Pseudomonas aeruginosa*, *Klebsiella pneumoniae*, and *Mycobacterium smegmatis*) or yeast (*Candida albicans*) cultures. In other fungal species (*Aspergillus flavus*, *Aspergillus terreus*, *Rhizopus oryzae*, and *Fusarium solani*), [^68^Ga]Ga-TAFC and [^68^Ga]Ga-FOXE showed lower levels of uptake compared with the level of uptake seen in *Aspergillus fumigatus*. [^68^Ga]Ga-FOXE but not [^68^Ga]Ga-TAFC showed uptake in *Staphylococcus aureus* culture. Both complexes showed no significant uptake in human lung cancer cells [116]. These results showed some but not a complete level of specificity of ^68^Ga-labeled siderophores for *Aspergillus fumigatus* infection.

A couple of modifications have been attempted to improve the in vivo biokinetics of ^68^Ga-labeled siderophores for possible clinical translation. Both [^68^Ga]Ga-TAFC and [^68^Ga]Ga-FOXE, the most successful radiolabeled siderophores, demonstrate intense renal retention precluding their use to assess renal involvement in IFD [115,116,117]. Attempts at structural modifications of ^68^Ga-labeled siderophores to reduce renal retention were unsuccessful [118]. IFD may be associated with severe tissue destruction requiring surgical excision. The conjugation of siderophores with fluorescent dye has been attempted for optical imaging [119,120]. The siderophore–fluorescent dye complex showed rapid uptake by *Aspergillus fumigatus* hyphae with the visualization of intracellular organelles from 5 min after application and lasting for more than two hours. The optical imaging of excised *Aspergillus fumigatus*-infected lung tissue obtained from rats injected with a siderophore–fluorescent dye complex displayed a high fluorescence signal congruent with ^68^Ga-labeled siderophore distribution in the same animal obtained on microPET/CT imaging [119]. The allure of using siderophore–fluorescent dye complexes in IFD lies in the potential for use to guide the extent of surgery.

Based on a comprehensive preclinical evaluation, ^68^Ga-labeled siderophores have been shown to demonstrate active trapping by *Aspergillus fumigatus*, most especially. The energy-dependent uptake by live pathogens can identify actual residual disease in a treated patient, which may help guide the duration of antifungal therapy. The prompt renal excretion of ^68^Ga-labeled siderophores with a high signal-to-noise ratio positions this radiopharmaceutical for potential application in IFD involving any organs of the body except the kidney. The successful complexation of siderophores to fluorescent dye for optical imaging makes them a promising tool for guiding tissue resection in patients requiring surgical intervention. Despite these promising results from preclinical studies, the application of radiolabeled siderophores for clinical IFD imaging is still being awaited. Iron overload may complicate repeated blood transfusion in immunocompromised hosts at risk of IFD. A high iron load can potentially decrease the sensitivity of radiolabeled siderophore imaging due to the reduced need for siderophore uptake by the pathogenic fungi [121]. In the preclinical study by Petrik et al., while rats pretreated with iron had a reduced intensity of radiolabeled siderophores at the sites of infection compared with rats that were not pretreated with iron, the level of difference did not reach statistical significance [117]. This indicates that more work is needed to determine the true impact of iron overload on radiolabeled siderophores by fungal agents.

#### 3.2.2. Targeting Fungal Cell Membrane/Cell Wall Synthesis

The synthesis of new membranes is a requisite process for growth in living cells. Cellular membrane synthesis is a common pathway inhibited in antimicrobial therapy. Ergosterol is an essential component of the fungal cell membrane. Ergosterol is synthesized from lanosterol in a reaction catalyzed by 14-α-demethylase, a cytochrome P450 enzyme. Azoles are a group of commonly used antifungals that inhibit 14-α-demethylase, preventing the formation of ergosterol, which eventually leads to fungal growth inhibition or cell death [122]. Fluconazole is one of the most used azoles for chemoprophylaxis and therapy of fungal diseases [123]. The radiolabeling of fluconazole to Technetium-99m (^99m^Tc) for SPECT imaging and Fluorine-18 for PET imaging has been described [124,125,126,127].

In a preclinical study by Lupetti et al., [^99m^Tc]Tc-fluconazole demonstrated in vitro stability in human serum [124]. The radiolabeling of fluconazole to ^99m^Tc did not influence its in vitro binding to *Candida albicans*. [^99m^Tc]Tc-fluconazole showed preferential binding to *Candida albicans* with a much lower binding affinity for *Aspergillus fumigatus*, human cells, *Staphylococcus aureus*, and *Klebsiella pneumoniae*. An in vivo biodistribution study in mice demonstrated a renal route of excretion for [^99m^Tc]Tc-fluconazole. In mice with *Candida albicans* infection induced in thigh muscles, [^99m^Tc]Tc-fluconazole accumulated in the site of fungal infection at a rate proportional to the viable pathogen level with an excellent target-to-background signal ratio. [^99m^Tc]Tc-fluconazole showed poor localization to the site of bacterial infection and sterile inflammation [100]. This study provided preliminary evidence supporting the feasibility of targeting fungal ergosterol synthesis for SPECT imaging [124]. No follow-up study to evaluate the utility of [^99m^Tc]Tc-fluconazole in human IFD has been published to date. Despite the attractions offered by the availability and cost-effectiveness of ^99m^Tc for the radiolabeling of pharmaceuticals, the lower resolution of the SPECT system compared with the PET system is a limitation to be borne in mind. The radiolabeling of fluconazole to ^18^F was, therefore, a welcome development [125,126,127]. Early studies of [^18^F]F-fluconazole reported a successful radiosynthesis of the tracer. [^18^F]F-fluconazole is highly lipophilic and undergoes hepatic metabolism, giving rise to high liver activity on PET imaging. This observation is an important limitation of [^18^F]F-fluconazole given that the liver is a common organ of involvement in IFD. Similarly, the utility of radiolabeled fluconazole may be limited to fungi species that are sensitive to this agent as fungal agents resistant to fluconazole may not accumulate the tracer significantly to allow for a sufficiently useful signal detectable by imaging at the sites of IFD.

Despite the limitation with [^18^F]F-fluconazole for IFD imaging, [^18^F]F-fluconazole PET imaging may find alternative applications in assessing the biodistribution of this radiopharmaceutical in different tissues and IFD involving different organs. In a human study evaluating the biodistribution of [^18^F]F-fluconazole, Fischman and colleagues utilized the data obtained from their study of the in vivo biodistribution of [^18^F]F-fluconazole to predict the adequacy of the dosing of fluconazole used in clinical practice [127]. According to their results, while 400 mg per day of fluconazole is sufficient for treating urinary tract and hepatosplenic candidiasis, it would be insufficient to treat candida osteomyelitis due to its limited penetration into bone tissues. Traditionally, clinical drug dosing is based on calculations obtained from animal studies of the drug. The study of the in vivo biodistribution of drugs in animals required multiple sampling of biological specimens and sacrificing animals to obtain the concentration of the drug in tissues. The use of the radionuclide technique for studying the in vivo biodistribution of drugs allows for the noninvasive exploration of the biokinetics of the drugs in humans without relying on extrapolated data from animal studies. Radionuclide techniques can be perfectly used for drug biodistribution studies and may be cheaper and more accurate than the currently used approaches for drug development [128,129,130].

A cell wall envelopes the fungal cell membrane, providing structural support to maintain cellular integrity. Caspofungin, an echinocandin, is an antifungal used in the treatment of invasive aspergillosis and candidiasis. It exerts its antifungal effect by inhibiting the formation of fungal cell walls. The radiolabeling of caspofungin to ^99m^Tc has been described [131]. The [^99m^Tc]Tc–caspofungin–tricarbonyl complex is stable in human serum with a hepatobiliary route of excretion. The [^99m^Tc]Tc–caspofungin–tricarbonyl complex demonstrated high accumulation at the sites of thigh muscle infection induced by *Aspergillus fumigatus* and *Candida albicans* in mice. Sterile inflammation induced by turpentine showed minimal tracer accumulation. These results showed that radiolabeled caspofungin is worth further exploration to determine its suitability for clinical translation. More studies are needed to define the performance of this radiotracer and its potential for clinical translation.

#### 3.2.3. Targeting Fungal-Specific Molecular Structures

The fungal cell has molecular structures that are unique to it. Targeting these structures for radionuclide imaging has the potential for fungal-specific imaging. A few radiopharmaceuticals targeting specific molecular structures of fungi have been synthesized and evaluated for their utility in IFD imaging with SPECT and PET techniques.

Ergosterol forms an integral part of the fungal cell membrane. Ergosterol is not found in the human cell membrane. It is, therefore, unique to the fungal cell membrane. Amphotericin B is a polyene agent with broad antifungal activity commonly used in the treatment of IFD. It exerts its antifungal activity by binding to fungal membrane ergosterol, leading to the formation of membrane pores that cause fungal cell death. The radiolabeling of amphotericin B to ^99m^Tc and ^68^Ga has been described [132,133]. In an in vitro study, [^99m^Tc]Tc-amphotericin B showed a time-dependent accumulation in *Aspergillus fumigatus*, reaching a peak at 60 min [133]. No significant [^99m^Tc]Tc-amphotericin B uptake was seen in normal human pulmonary artery endothelial cells or *Staphylococcus aureus*. In mold infection, the spore form of the organism is the infective form, while the hyphal form is the tissue-invasive form. It is, therefore, important to differentiate the spore form, which may represent mere colonization from the hyphal form of the organism, which causes disease. [^99m^Tc]Tc-amphotericin B accumulates in tissue culture infected with the hyphal but not spore forms of *Aspergillus fumigatus* and *Aspergillus arrhizus* [133]. Interestingly, fungal species known to be resistant to amphotericin B, including *Aspergillus terreus* and *Cunninghamella bertholletiae*, also accumulated [^99m^Tc]Tc-amphotericin B significantly, indicating that all that is necessary for this radiopharmaceutical to accumulate at the site of IFD is the presence of ergosterol in the causative fungal agent membrane and not the sensitivity of the pathogen to amphotericin B [133]. The results of the experiments with [^68^Ga]Ga-amphotericin B were largely similar to those obtained for [^99m^Tc]Tc-amphotericin B [133]. The in vivo behavior of these radiopharmaceuticals is yet to be comprehensively evaluated. A preliminary in vivo study in mice shows significant [^99m^Tc]Tc-amphotericin B in *Aspergillus fumigatus* and *Candida albicans* infections [132]. The accumulation of [^99m^Tc]Tc-amphotericin B at the site of sterile inflammation was minimal [132].

A potential limitation to the clinical application that may be experienced with these agents is the known affinity of amphotericin B for cholesterol present in the human cell membrane [134]. This affinity forms the basis of the nephrotoxicity of amphotericin B due to its accumulation in renal tubular cells [134]. In the in vivo study of [^99m^Tc]Tc-amphotericin B described above, the radiopharmaceutical demonstrated a renal route of excretion with minimal renal activity at 3 and 6 h post tracer injection. Results from the clinical study of the behavior of radiolabeled amphotericin B are still being awaited.

#### 3.2.4. Targeting Hyphal-Specific Antigen

The utility of the radionuclide technique in discriminating between the infective hyphae and the inactive spores of *Aspergillus* species has been explored further using radiolabeled antibodies targeting *Aspergillus* mannose proteins that are only expressed during active hyphal growth [135,136]. In the study by Rolle et al., JF5, a monoclonal antibody against *Aspergillus* mannose proteins, was successfully radiolabeled with copper-64 (^64^Cu) using DOTA as the chelator [135]. [^64^Cu]Cu-DOTA-JF5 demonstrated in vitro stability in human serum. PET imaging demonstrated a significantly elevated uptake of [^64^Cu]Cu-DOTA-JF5 in the lungs of mice infected with *Aspergillus fumigatus* compared with the lungs of mice infected with *Streptococcus pnuemoniae* or *Yersinia enterocolitica*. Besides the uptake in infected lungs, high activity of [^64^Cu]Cu-DOTA-JF5 was also seen in the blood pool, liver, spleen, and kidneys [135]. These results indicate the feasibility of targeting mannose proteins of *Aspergillus* that are specifically and abundantly expressed during rapid hyphal growth. Despite its promise, there are particular concerns regarding the clinical translation of this agent. Firstly, monoclonal antibodies are associated with human anti-mouse antibody (HAMA) reaction, which may prevent repeated administration of the agent. Secondly, the background activity in the blood pool and multiple visceral organs may not only mask the detection of disease in contiguous organs but also preclude the use of this agent for assessing IFD involvement in these organs with high physiologic tracer uptake. These concerns were addressed by the same authors in a subsequent study where they used the humanized form of JF5 (hJF5) for radiolabeling to ^64^Cu using NODAGA instead of DOTA as the chelator [136]. The use of a humanized monoclonal antibody can reduce the risk of HAMA, allowing for repeated administration, especially in the context of treatment response assessment. Significant background activity, especially in the cardiovascular system, remained. This latter limitation is related to the long circulating time of a whole antibody labeled with a radionuclide with a relatively long physical half-life. While this method holds much promise for clinical translation, more work needs to be performed to optimize its performance.

#### 3.2.5. Targeting Fungal Cell Wall Chitin

Chitin is another component of the fungal cell wall that is not present in mammalian or bacterial cells. Chitinases are glycosyl hydrolase enzymes that break down chitin. Siaens et al. have described the radioiodination with iodine-123 (^123^I) of a modified chitinase obtained from the bacterium *Serratia marcescens* [137]. [^123^I]I-chitinase demonstrated intense binding to *Aspergillus fumigatus* and *Candida albicans*. There was no significant binding of [^123^I]I-chitinase to bacterial cells (*Staphylococcus aureus* or *Escherichia coli*) or human cells (erythrocytes or leucocytes). In an in vivo biodistribution study in mice, the stomach and urinary bladder had the highest activity, with some activity in the thyroid gland as well. Scintigraphic imaging performed 24 h post tracer injection confirmed [^123^I]I-chitinase specificity for fungal disease with a high tracer accumulation in the stomach, thyroid gland, and urinary bladder. The intense activity seen in the stomach and thyroid gland results from the dehalogenation of the radiopharmaceutical in vivo, a common phenomenon with radio-halogenated proteins. ^123^I is an expensive radionuclide due to its production from a cyclotron. Siaens and colleagues have further described the radiolabeling of another chitinase molecule with ^99m^Tc for scintigraphic imaging [138]. The specificity of [^99m^Tc]Tc-chitinase for fungal infection was also demonstrated in this subsequent study. Like most other fungal-specific radiopharmaceuticals, no clinical data on radiolabeled chitinase for IFD imaging are available yet.

#### 3.2.6. Targeting Fungal Ribosomal RNA

Fungal ribosomal ribonucleic acid (rRNA) is an attractive molecular target that can be explored to detect the presence of a specific fungus in vivo. The base sequence of the rRNAs of many fungi is known, rRNA is present in the fungi in abundance, and their expression level is reasonably constant over time. These features combine to make rRNA an attractive target for the detection of a pathogen in vivo. Oligonucleotide probes that bind to the rRNA of specific bacteria and fungi have been developed for the in vitro identification of these organisms [139]. Oligonucleotide probes with a radionuclide tag can be used for the in vivo identification of pathogenic fungi using SPECT and PET techniques. Wang and colleagues radiolabeled morpholino oligomers (MORFs), deoxyribonucleic acid (DNA) oligomers that bind to their complementary DNA or RNA with high affinity, for SPECT imaging of invasive aspergillosis in mice [116]. The authors confirmed the specific binding of [^99m^Tc]Tc-MORF probes to the RNA of *Aspergillus fumigatus*, *Aspergillus flavus*, and *Candida albicans*. In a biodistribution study, [^99m^Tc]Tc-MORF probes cleared promptly from the circulation. The organ with the highest retention of [^99m^Tc]Tc-MORF probes was the kidney due to the renal route of excretion of the radiopharmaceuticals. There was a significantly higher accumulation of [^99m^Tc]Tc-MORF probes in the lungs of infected mice compared with healthy controls [140]. This study opens a novel opportunity worthy of further exploration for possible application in the evaluation of IFD. This further exploration of the suitability of this tracer for IFD imaging is needed to establish its potential for clinical translation and the limitation of its applications.

### 3.3. Non-Specific Antimicrobial Peptides

In addition to radiolabeled anti-fungal drugs targeting specific molecular structures of the fungi, other non-specific antimicrobial peptides have been explored for their possible application as noninvasive probes for IFD imaging [26,141]. Ubiquicidine 29–41 (UBI 29–41) radiolabeled with ^99m^Tc for SPECT or ^68^Ga for PET imaging have been extensively used for pyogenic skeletal and soft tissue infections [142,143,144]. [^99m^Tc]Tc-UBI 29–41 has been reported to accumulate at sites of *Aspergillus fumigatus* and *Candida albicans* infections [124,145]. [^99m^Tc]Tc-UBI 29–41, like other non-specific radiolabeled antimicrobial peptides and proteins including [^99m^Tc]Tc-lactoferrin and [^99m^Tc]Tc-immunoglobulin G, cannot discriminate between bacterial and fungal infections [124,145]. They, therefore, have a limited role to play in the specific targeting of IFD using radionuclide techniques.

## 4. Conclusions and Future Perspectives

In the immunocompetent host, the functional host immune system can resist tissue invasion by fungi. Fungal organisms grow and invade deep host tissue in the atmosphere of immune suppression, causing IFD. IFD contributes significantly to the morbidity and mortality of immunocompromised hosts, including solid organ transplant recipients, hematopoietic cell transplant recipients, patients with hematologic malignancies, HIV-infected patients, and many more. The list of immunocompromised hosts at an increased risk of IFD is growing, with the latest addition being SARS-CoV-2-infected COVID-19 patients. Radionuclide imaging with SPECT and PET holds great promise for use in the identification and treatment response assessment of IFD. A growing body of evidence suggests that [^18^F]FDG PET/CT is superior to the currently recommended morphologic imaging with CT and MRI for the detection and treatment response assessment of IFD. The lack of specificity of [^18^F]FDG PET for IFD has led to a great interest in developing more specific probes targeting molecular structures or metabolic pathways unique to pathogenic fungi. Several preclinical studies have evaluated these specific probes, and evidence to support their clinical translation is still being awaited.

Despite the superior performance of [^18^F]FDG PET/CT for lesion detection and early response assessment in IFD compared with morphologic imaging by CT and MRI, [^18^F]FDG PET/CT is still not included in guidelines as a recommended modality for these indications. To address this, more work is needed to provide more robust evidence to justify the inclusion of [^18^F]FDG PET/CT in clinical practice guidelines of IFD management. Large prospective multicenter studies addressing the impact of the superior lesion detection of [^18^F]FDG PET/CT in IFD over morphologic imaging on treatment outcome are needed. The cost-effectiveness of the inclusion of [^18^F]FDG PET/CT into the treatment algorithm of IFD is needed. Evidence needs to be synthesized to guide the timeline of [^18^F]FDG PET/CT application for response assessment in patients with IFD.

Many probes with potential for specific fungal targeting have been explored at the preclinical level. None of these has been translated to clinical application, suggesting residual concern regarding their performance. The development of animal models of different types of IFD reflecting the different stages of the disease (from mild to severe) is a mandatory first step for the rational preclinical evaluation of candidate fungal-specific radionuclide probes. Radionuclide techniques hold promise for use in drug development using radiolabeled antifungal agents for dynamic PET imaging. The recently introduced total-body PET system can contribute significantly to the use of radionuclide techniques for drug development as it allows the determination of the pharmacokinetics of drugs in different lesions and tissues anywhere in the body in real time.

## Data Availability

Not applicable.

## Abbreviations

AIDS:	Acquired immunodeficiency syndrome
AMP:	Anti-microbial peptides
BTK:	Bruton tyrosine kinase
CLRs:	C-type lectin receptors
CT:	Computed tomography
DAMPs:	Danger-associated molecular patterns
DCs:	Dendritic cells
[^18^F]FDG:	Fluorine-18 fluorodeoxyglucose
GvHD:	Graft-versus-host disease
HCT:	Hematopoietic cell transplantation
HIV:	Human immunodeficiency virus
IFD:	Invasive fungal disease
MORFs:	Morpholino oligomers
MRI:	Magnetic resonance imaging
NETs:	Neutrophil extracellular traps
NK:	Natural killer
PAMPs:	Pathogen-associated molecular patterns
PET/CT:	Positron emission tomography/computed tomography
PJP:	*Pneumocystis jirovecii* pneumonia
PRRs:	Pathogen recognition receptors
SIT:	Siderophore–iron transporter
SOT:	Solid organ transplant
SPECT:	Single-photon emission tomography
TAFC:	Triacetylfusarinine C
TLG:	Total lesion glycolysis
TLRs:	Toll-like receptors
